# Barriers and facilitators to recognize and discuss depression and anxiety experienced by adults with vision impairment or blindness: a qualitative study

**DOI:** 10.1186/s12913-021-06682-z

**Published:** 2021-07-28

**Authors:** Edine P. J. van Munster, Hilde P. A. van der Aa, Peter Verstraten, Ruth M. A. van Nispen

**Affiliations:** 1 Ophthalmology, Amsterdam UMC, Vrije Universiteit Amsterdam, Amsterdam Public Health Research Institute, Amsterdam, The Netherlands; 2 Expertise Innovation Knowledge, Robert Coppes Foundation, Vught, The Netherlands

**Keywords:** Vision loss, Vision impairment, Blindness, Depression, Anxiety, Detection

## Abstract

**Background:**

Depression and anxiety are highly prevalent, but often unrecognized in adults with vision impairment (VI) or blindness. The purpose of this study was to explore visually impaired and blind adults’ views on facilitators and barriers in recognizing and discussing mental health problems.

**Methods:**

Semi-structured interviews, based on the Integrated Model for Change, were conducted with 16 visually impaired or blind adults receiving support from three Dutch low vision service organizations. Interview data was analyzed using the framework approach.

**Results:**

Participants perceived their focus on practical support with regard to their VI, lack of mental health literacy, and misattribution of symptoms of depression or anxiety as barriers for recognizing mental health problems. With regard to discussing mental health problems, they perceived difficulties in acknowledging their VI and mental health problems due to feelings of vulnerability and inequality. Participants mentioned that their social support system and healthcare providers (could) facilitate them in recognizing and discussing mental health problems. However, participants thought that healthcare providers currently often lacked the knowledge, skills and attitude to recognize and discuss this topic with their clients.

**Conclusion:**

Our findings suggest that visually impaired and blind adults may experience several barriers to recognize, acknowledge and discuss mental health. Healthcare providers and social support systems seem essential for them in reducing these barriers. However, there might be a mismatch between the needs of visually impaired and blind adults and healthcare providers’ knowledge, skills and attitude. Training healthcare providers may improve detection of depression and anxiety in adults with VI or blindness, and enhance clinician-patient communication on mental health.

**Supplementary Information:**

The online version contains supplementary material available at 10.1186/s12913-021-06682-z.

## Introduction

Current estimates of people who are blind or have moderate or severe vision impairment, are around 338 million, and are expected to increase to 535 million people by the year 2050 [1]. Vision impairment (VI) and blindness may have a direct effect on physical dysfunction and limitations in daily life activities, and may lead to increased symptoms of depression and anxiety. About 5 % of adults with VI or blindness has a major depressive disorder and about 7 % has an anxiety disorder [2]. Moreover, one in three experience subthreshold symptoms of depression and/or anxiety [2–5], indicating clinically significant symptoms but no actual disorder. Based on these prevalence estimates, approximately 100,000 visually impaired or blind adults living in the Netherlands experience subthreshold depression and/or anxiety [6, 7]. These prevalence estimates are significantly higher compared to the general population [2]. In visually impaired and blind adults, having (subthreshold) depression can lead to decreased health-related and vision-related quality of life and visual functioning [8]. Less is known about the effects of (subthreshold) anxiety in adults with VI or blindness [9]. However, they more often experience anxiety related to specific places or situations and social situations compared to normally sighted peers [2]. An early treatment approach is recommended to reduce negative influences on quality of life and to prevent development of a full blown disorder.

Despite effective mental health treatments available for people with VI or blindness [10], more than half do not receive any mental health support for depression or anxiety [11–13]. Different barriers for receiving treatment are expressed by visually impaired and blind adults. A former study showed that they often experience a lack of knowledge about symptoms and treatment possibilities, followed by not wanting to rely on others [11]. Symptoms of depression and anxiety seem to be systematically overlooked by visually impaired and blind adults themselves, but also by others.

Healthcare providers, i.e. eye care practitioners and low vision service workers, often do not recognize depression in visually impaired and blind adults. From the perspective of healthcare providers, this may be due to their focus on physical health instead of psychological health [14]. A lack of confidence in eye care practitioners’ knowledge and skills seems to limit them in recognizing symptoms of depression in adults with VI or blindness [15]. Other examples of barriers experienced by eye care practitioners and low vision service workers are lack of training in recognizing depression, absence of standard procedures within their organizations to detect depression, limited time and high workload [14, 16]. Moreover, many eye care practitioners believe visually impaired and blind adults themselves create barriers: denial and a defensive attitude are the most common barriers mentioned [14]. Healthcare providers believe reluctance to discuss depression might be due to difficulties in communication, social stigma related to depression, or perceived negative consequences of acknowledging depression [14, 15].

While a few studies investigated barriers from the healthcare providers’ perspective, so far, no in-depth research has been performed to explore the perspective of visually impaired and blind adults. In addition, previous research focused on detection of depression, leaving anxiety underexposed, while prevalence estimates of anxiety are high as well [2]. Therefore, this study aimed to explore the process of recognizing and discussing depression and anxiety in visually impaired and blind adults. Barriers and facilitators that contribute to the identification and discussion were explored, with special attention on the healthcare provider’s role.

## Methods

### Study design and participants

Visually impaired and blind adults who experienced depression or anxiety were recruited to participate in this qualitative study. The following eligibility criteria were used: (1) 18 years and older; (2) current or history of (subthreshold) depression and/or anxiety; (3) moderate visual impairment, severe visual impairment or blindness according to the World Health Organization (WHO) criteria [17]. Adults with severely impaired cognitive abilities or minimal understanding of the Dutch language were excluded from participation. The six-item screener, a short version of the Mini Mental State Examination, was used to measure participants’ cognitive abilities with scores lower than three indicating severely impaired cognitive abilities [18].

Participants were purposively recruited from three Dutch low vision service organizations. These low vision service organizations provide multidisciplinary services to support people in dealing with their vision impairment and blindness. Mostly outpatient services are provided, such as prescribing low vision aids, mobility training and counseling, but also long and short term inpatient care is provided. Psychologists working at the low vision service organizations were asked to select eligible participants based on the clients’ medical history, approach them by telephone or during face-to-face meetings, offer them a written information letter and informed consent form, and answer questions if applicable. All participants provided written consent. One participant who consented to participate dropped out due to declining mental health.

### Data collection

Semi-structured face-to-face interviews with individual participants were performed by the first author (EvM), who worked as a researcher at one of the low vision service organizations, but had no prior relationship with the participants. Interviews were conducted at the participant’s home, except for two interviews that were conducted at the low vision service organization. Participants were allowed to bring a trusted person to the interview, which occurred during two interviews. Interviews lasted between 27 and 85 min (mean = 64 min), were audio-recorded and transcribed verbatim. Immediately after each interview field notes were completed and recorded emotions expressed by the participant, descriptions of concrete situations provided, statements about difficulty remembering, reflections on own experiences, general perspectives, and experiences of the interviewer.

### Theoretical framework

The Integrated Change model of de Vries et al. (e.g. the I-Change model) was used as a theoretical framework to develop the interview guide (see Additional file 1), and to analyze barriers and facilitators for the detection and discussion of depression and anxiety [19]. The I-Change model is an integrative model using several scientific models about social cognition, and explains motivational and behavioral change. I-Change is used in research about healthcare utilization from both healthcare provider and client perspective [20, 21]. According to the I-Change model, behavior is determined by someone’s intention, which is influenced by motivational factors (i.e. attitude, social influence and self-efficacy). In turn, these motivational factors are determined by awareness factors (i.e. knowledge, cues to action and risk perception) and predisposing factors. In these predisposing factors, personal factors and sociocultural factors can be determined [19].

### Analyses

Thematic analysis of the interview data was performed to describe and understand barriers and facilitators. I-Change model determinants were used as the coding framework [22]. All analyses were performed by two researchers (EvM, HvdA) using Atlas.ti8 software. The first step of analyzing the interview data involved open coding to help the researchers get familiar with the data. Several interviews were coded and consensus was reached, based on which the codebook was developed. Second, the codebook was used to analyze all interviews. Third, codes were clustered into subthemes. It was concluded that the last interview lacked new subthemes indicating that data saturation may have been reached. Subsequently, subthemes were summarized into main themes and assigned to domains based on consensus between the two researchers. Finally, field notes were checked to determine the degree of incompleteness due to lack of reflective ability or the ability of participants to look at their own situation from a distance, in a more general perspective.

## Results

Sixteen adults with VI or blindness (44 % male) participated in this study. Mean age was 60 years and ranged between 33 and 91 years. Participants’ medical files showed different diagnoses as cause of their VI (Table 1). In six participants comorbidities, such as hearing loss, autism spectrum disorder or physical complaints, were present.

**Table 1:** Participant characteristics (*N* = 16)

Patient characteristics	N (%)	Mean (SD)	Median [range]
**Male gender**	7 (43.8%)		
**Age (in years)**		59.8 (14.4)	58.0 [33 – 91]
**Moderate – severe vision impairment**^**a**^	4 (25.0%)		
**Blindness**^**a**^	12 (75.0%)		
**Acquired VI (age of onset)**	12 (75.0%)	40.5 (20.5)	35.5 [12 – 78]
**Eye disease**			
Retinal detachment	4 (25.0%)		
Optic nerve disease	4 (25.0%)		
Macular degeneration	3 (18.7%)		
Other retinal disease	3 (18.7%)		
Other	2 (12.5%)		
**Symptoms of depression in the past**	13 (81.3%)		
**Symptoms of anxiety in the past**	7 (43.8%)		
**Current symptoms of depression/anxiety**	5 (31.3%)		

*SD* standard deviation, *VI* visual impairment

^a^according to World Health Organizations (WHO) criteria

### Barriers and facilitators

Main themes and subthemes identified through the inductive process were mapped to domains within the I-Change model. These were: (1) predisposing factors, (2) environmental factors, (3) awareness related factors, and (4) motivational factors. Two domains were added based on the input that was gathered: (5) social support system and (6) healthcare provider’s role. Table 2 represents all facilitators and barriers gathered within these domains, their themes and sub-themes.

**Table 2:** Themes and sub-themes in detection and discussion of (subthreshold) depression and anxiety

Domain	Theme	Sub-theme
1. Predisposing factors	Coping	• Coping strategies (+, -) • Internal locus of control (+, -) • Personality traits (+, -)
2. Environmental factors	Acquired care	• Receive care from low vision service organization while experiencing symptoms (+, -) • Experiences with low vision service organization (+, -)
	Social inclusion	• Stigma related to VI (-) • Feelings of inequality (-)
3. Awareness related factors	Risk perception	• Impact VI on mental health (+, -) • Self-assessed severity of symptoms (+, -) • Need for help (+, -)
	Detection	• Recognition of psychological complaints or changes in behavior (+) • Focus on VI (-) • Misattribution of symptoms of depression or anxiety (-)
	Knowledge	• Knowledge of mental health interventions (-) • Limited information collection due to VI (-)
4. Motivational factors	Attitude	• Attitude towards discussion (+, -) • (Dis)advantages of discussion (+, -)
	Willingness to discuss	• VI complicates discussion(-) • Self-confidence on discussion (+, -)
5. Social support system	Informal emotional support	• Recognition and discussion by social support system (+, -) • Guidance and encouragement from social support system (+, -) • Indifference and incomprehension impact VI on mental health (-)
	Network size	• Number of social contacts (-)
6. Healthcare provider’s role	Focus of healthcare provider	• Focus on practical rehabilitation VI (-) • Attention impact VI on mental health (+, -)
	Formal support	• Referral to healthcare provider with knowledge VI (+) • Discuss mental health/current symptoms (+, -) • Help-seeking (+, -) • Transfer knowledge (-)
	Expertise of healthcare provider	• Knowledge healthcare provider (-) • Skills healthcare provider (-) • Attitude healthcare provider (-) • Relationship with adult with VI (+)

*VI* Visual Impairment

+ facilitator, - barrier

### Predisposing factors

Participants used various ways to cope with their mental health problems. More than half of them mentioned a passive or ineffective coping strategy, such as denial and overcompensation by wanting to show others that their VI had no or a minimal effect on their life. Active problem solving was addressed as an often used coping strategy by a few participants. Some of them tried to solve their problems relying on their own resources, while others took the initiative to ask for help, most often from their general practitioner (GP). *“My husband cannot fix this. A guide dog cannot solve this. I am the only one who can solve this, but I have to act now. Therefore, I went to my general practitioner (female, aged 49, blind).”*

### Environmental factors

A few participants mentioned that receiving care from low vision service organizations increased their likelihood of discussing depression or anxiety, in any stage of the symptoms, just because they had access to a low vision service worker. In addition, having a VI changed some participants’ perspectives on social inclusion due to perceived stigma and an experienced lack of equality. They felt that their VI made them different, vulnerable and unequal to others, and that discussing mental health problems would increase those feelings. One participant mentioned: *“I feel like they are looking down on me, because I am already different from everyone else. (…) If I can just participate in society in a normal way or if everyone sees me as a normal person, that is already so different. Then mental health problems become less uncomfortable and more negotiable (female, aged 41, blind).”*

### Awareness

In the beginning almost all participants focused on the practical implications of their VI, and therefore failed to acknowledge its impact on their mental health. In addition, half of the participants mentioned misattribution of symptoms limited their recognition. They thought symptoms such as having low energy, physical complaints and having less interest in activities were related to their old age, their personality, medicines they used, a previous accident or their VI instead of acknowledging them as mental health problems.

*Whenever I feel like something is wrong with me, I blame it on the car accident I had 34 years ago. I do not know if that makes sense. I mean, old age comes with deficiencies (female, aged 58, low vision).*

Later on in the coping process, participants became aware of the significant impact of the VI on their mental health. They often believed that feelings of vulnerability and inequality that they experienced based on their VI aggravated their mental health problems. They also linked coping with permanent loss, or future losses in progressive eye diseases, to depression and anxiety. One participant explained: *“Vision loss is bigger than just losing your sight. There is so much more you cannot do anymore, which makes you feel worthless and changes you as a person (female, aged 49, blind).”*

Being unaware of possibilities for receiving mental health support was often mentioned as a barrier for discussing symptoms, especially within low vision service organizations. Two participants still lacked knowledge about where to find appropriate care. Some participants explained that their reduced ability to collect visual information may have caused this lack of knowledge: *“The general practitioner’s waiting room is full of posters. If you are a good sighted person waiting, you can look around and can be triggered to investigate a subject further on the internet. As a blind person you just happen to hear it or need to think of it yourself (female, aged 41, blind).”* Because of the decreased ability to receive and collect information, participants stressed the importance of healthcare providers, i.e. eye care practitioners, general practitioners and low vision service workers, to provide appropriate information. This information should be about the increased risk of depression and anxiety in people with VI and blindness, and about possibilities for support.

### Motivation

Both advantages and disadvantages of discussing feelings of depression and anxiety with a healthcare provider emerged. The prospect of receiving support was mentioned as an advantage. Participants felt that tailored support could help them comprehend and improve their situation, and help them feel in control again. Disadvantages included fear of further deterioration of mental health by discussing it, fear of potential changes in daily life, and the need to acknowledge their VI. One participant explained: *“The moment I was going to discuss it with a psychologist, I had to admit something was wrong. I miss something (vision) and I have to adjust my life accordingly. I wasn’t ready until last year (female, aged 47, blind).”*

 Participants often considered their VI made discussing mental health with a healthcare provider more difficult. Several of them indicated that their VI made it difficult to open up about their mental health problems, because they had to acknowledge their disability and deal with its consequences. Also, they had to open up about two subjects that made them feel vulnerable. One participant mentioned VI could also decrease trust in others, because it limits interpretation of body language. Another participant referred to having depression as an extra burden on top of his VI: *“People without vision loss do not struggle with a visual impairment. Therefore, they have the capacity and time to put energy in other things, like feelings of depression (male, aged 33, blind).”*

### Social support system

Informal emotional support was indicated as a significant facilitator in recognizing and discussing depression and anxiety. More than half of the participants felt guided by a loved one, who helped them to recognize the symptoms of depression or anxiety, and encouraged them to discuss it with a healthcare provider. However, some participants lacked informal support or received more practical solutions, e.g. write feelings down or get a guide dog. Some of them also expressed their loved ones’ incomprehension of the impact of VI on mental health: *“It was the beginning of us growing apart. She (partner) literally shrugged her shoulders and said ‘You’ll get over it.’ As if it was a common cold (male, aged 56, blind).”* One participant mentioned that VI could limit the size of a person’s social network due to loss of daily activities (e.g. losing their job or decrease in social activities), and therefore might leave them with fewer people that are able to provide informal support.

### Healthcare providers

Participants expressed the importance of the healthcare provider’s role in their recognition and willingness to discuss symptoms. They mentioned that eye care practitioners and GPs not often linked the VI with mental health problems and almost never discussed mental health. Nevertheless, participants were positive about their referrals to low vision service organizations, because they expected healthcare providers with knowledge of VI would understand their situation. However, only half of the participants mentioned a low vision service worker discussed mental health after referral, and if discussed, always by social workers or counsellors. In addition, low vision service workers often focused too much on the practical side of low vision rehabilitation and had little attention for the impact of VI on mental health. *“VI definitely has an impact. Actually, there are institutions that can help you deal with using an iPad or they tell you that you can no longer drive a car. But in that case your state of mind is ignored (male, aged 80, low vision).”*

Participants mentioned that healthcare providers should have a constant focus on possible mental health problems in people with VI, from the first diagnosis until the end of rehabilitation, and anticipate on mental difficulties in the future. “*In retrospect, I think it makes sense that healthcare providers confronted me with the fact that my vision is deteriorating and I was probably unable to drive a car in the future. (…) Also acknowledge that it can hurt and make you feel anxious (female, aged 77, low vision).”* Eye care practitioners and GPs should be aware of both the physical and emotional impact of VI, and the opportunities for support. Participants stressed the importance of follow-up care to check upon adults with VI or blindness, and referrals to low vision service organizations in an early stage. *“I think when ophthalmologists diagnose permanent vision loss, it should trigger them to start providing care (male, aged 64 blind).”* Participants recommended that healthcare providers invite them to talk about mental health problems and transfer their knowledge about different aspects of depression and anxiety in relation to the VI, such as prevalence rates, possible symptoms that may be experienced and opportunities for receiving support.

However, participants thought that healthcare providers, especially GPs, often lacked knowledge, confidence, skills, expertise and the proper attitude to detect and discuss depression and anxiety. They mentioned some healthcare providers lacked skills in empathizing with visually impaired and blind adults concerning these symptoms. GPs and eye care practitioners seemed to be unaware of the impact of VI on mental health, and have difficulty referring adults with VI or blindness to the appropriate care, and low vision service workers tended to have difficulty linking the impact of VI with mental health problems as well. Moreover, participants assumed a lack of critical attitude in low vision service workers because they often focused on practical solutions regarding VI, and occasionally trusted participants’ statements about having a good mental health too easily. Participants proposed that healthcare providers consider complaints as an aspect of depression or anxiety, and integrate mental health in their routine care, for example by using a screening instrument. *“A general practitioner should check some things in adults with VI by default, such as energy, activities and mood. Ask how everything is going and if necessary: provide a referral (male, aged 33, blind).”* Finally, participants indicated a longer, persistent, equal and trustworthy relationship with their healthcare provider as facilitating. According to participants, healthcare providers can establish this by sharing personal stories and considering themselves equal to visually impaired and blind adults.

## Discussion

The aim of this study was to explore facilitators and barriers in the detection and discussion of depression and anxiety in visually impaired and blind adults. This study uncovered several important facilitators and barriers in recognizing, acknowledging and discussing mental health that might be specific for visually impaired and blind adults. Their social support system and healthcare providers seemed important facilitators in this process. Our findings may help healthcare providers, low vision service organizations, hospitals, GP practices and policy makers to understand the needs of visually impaired and blind adults, and adjust current care accordingly.

Participants seemed to experience difficulties in recognizing their mental health problems. Some indicated this was due to limited knowledge about the impact of VI on mental health and treatment possibilities. Limited knowledge on mental health (care) is more often reported as a barrier for help-seeking in adults with VI or blindness than in the general population [11, 23, 24]. This may be caused by the limited abilities of people with VI or blindness to obtain processable information, which can lead to low health literacy [25, 26], i.e. the ability to “obtain, process and understand basic health-related information and services to make appropriate health decisions” [27]. Health literacy seems an important facilitator in help-seeking for mental health problems [28]. People with VI or blindness might face specific barriers in obtaining health-related information because it is inaccessible (e.g. posters in a waiting room or information on a website). This emphasizes the importance of using accessible and tailored ways of informing people with VI or blindness on mental health problems and treatment possibilities, e.g. during contacts with an experienced healthcare provider or via audio recordings on a website. Another important reason for difficulty in recognizing mental health problems may be a misattribution of symptoms. Some symptoms of depression and anxiety, such as loss of daily activities, poorer self-care and fatigue, are often seen in people with VI or blindness [29–31], but can also be symptoms of mental health problems as they are highly prevalent in this population. It is warranted to educate visually impaired and blind adults about their increased risk of mental health problems, symptoms to recognize depression and anxiety and possibilities for support, also called psychoeducation, at the start of the eye disease and again if they qualify for low vision services.

Participants acknowledged that depression and anxiety are highly prevalent in people with VI or blindness. However, they seemed to encounter difficulties in being open about their mental health problems. Previous studies in adults with VI or blindness confirm this and sometimes even find that they tend to deny psychological distress [32, 33]. Some participants indicated that visually impaired and blind adults need to acknowledge VI or blindness before they can initiate discussing mental health. Nevertheless, mental health problems often occur when someone refuses to acknowledge their disability. People with VI or blindness can be recurrently confronted with their loss, because new situations and new problems keep redefining their loss [34], for instance not being able to see a newborn grandchild can result in another confrontation with being visually impaired or blind. This suggests that adults with VI or blindness need to adapt to and acknowledge their VI repeatedly during their lives. Healthcare providers should be aware of these reoccurring confrontations with loss of vision that may lead to mental health problems. Moreover, feelings of vulnerability, inequality and decreased trust in others seems to limit visually impaired and blind adults to discuss mental health. Many visually impaired and blind adults experience self-stigma on both VI and mental health problems, which may exacerbate these difficulties. Self-stigmatization is the result of internalizing negative stereotypes and may prevent them from seeking help and receiving treatment [35–37]. Psychoeducation can potentially reduce self-stigma [38], which emphasizes the importance of healthcare providers to provide information about the link between mental health problems and VI to help visually impaired and blind adults to open up about mental health problems.

 Participants often mentioned that support helped them to recognize and discuss mental health problems. Our findings showed that an active problem solving coping strategy seemed to assist visually impaired and blind adults in being able to discuss symptoms with a healthcare provider. However, literature showed that they often report a loss of control, low self-esteem and increased dependency on others for many daily activities [39–41]. Especially, adults with VI or blindness with an avoidant coping style seem to experience mental health problems [42] and people with mental health problems seem to have more difficulty in using adaptive coping strategies [43]. Therefore, support seems to be important in visually impaired and blind adults. Support consists of instrumental support (e.g. assisting with tasks of daily living) and emotional support (e.g. affective support) [44]. Strong informal emotional support is associated with help-seeking in mental health problems [45]. However, adults with VI or blindness more often receive instrumental support than emotional support, and most often responsibilities for providing support lies with their family members [46]. Participants experienced different levels of emotional support, that may be explained by adaptation to vision loss. Vision loss is associated with possible isolation from the family, changes in roles and responsibilities between family members, and burden within family members [47–49]. Therefore, some social support networks might have focused on providing instrumental support or had limited resources to provide emotional support.

Healthcare providers, i.e. eye care practitioners, general practitioners and low vision service workers, could help visually impaired and blind adults to recognize and discuss mental health problems as well. It seems important that healthcare providers understand the impact of VI on mental health, start a conversation about mental health and share knowledge about prevalence and symptoms of mental health problems. A previous study in women with VI confirms healthcare providers’ importance in achieving health literacy [25]. However, healthcare providers often seem to focus on VI, which is consistent with previous studies [14]. Participants also expressed the need of receiving information about the impact of VI on mental health and sometimes questioned the expertise (e.g. knowledge, skills and attitude) of healthcare providers. Nevertheless, only a quarter of ophthalmic and low vision service workers provides education and information for suspected depression [16]. In addition, they often report a lack of confidence in knowledge and skills as barriers to depression management in adults with VI or blindness [15, 16]. These barriers may have limited healthcare providers in providing information about mental health and treatment options, but also in starting a conversation about depression or anxiety. A possible explanation for the lack of critical attitude might be that healthcare providers think visually impaired and blind adults are often reluctant to discuss mental health [14, 15]. Therefore, there might be a mismatch between the needs of visually impaired and blind adults and healthcare providers’ abilities, resulting in underrecognition of mental health problems.

### Strengths and limitations

As far as we know, this study is the first to explore potential barriers and facilitators in recognizing and discussing mental health from the perspective of visually impaired and blind adults. The qualitative design allows us to understand the actual experiences of this fragile population in discussing this highly prevalent problem. Use of the I-Change model to create a comprehensive interview guide adds robustness to our methods and increased reliability of the results. Including a heterogeneous group from different gender, age groups and with various ophthalmic diagnosis and comorbidities contributes to exploring a broad picture of experienced barriers and facilitators.

Despite possible generalizability to adults who do not receive support from a low vision service organization, the results might lack generalizability due to a small sample size, and the lack of diversity in for instance cultural differences, cognitive abilities and (in)experience in discussing mental health problems. The retrospective design of the study allowed participants to share their experiences throughout the process of recognizing and discussing mental health problems. However, it could also have resulted in inaccuracy or incompleteness of recollection, also called recall bias [50]. In addition, some participants showed difficulties to indicate in concrete terms what helped or limited them to recognize, acknowledge and discuss mental health problems. In two interviews a trusted person was present, which could have influenced the results, for instance because the participant did not feel free to answer all questions honestly. Moreover, participants’ psychiatric or physical comorbidities may have aggravated the experienced barriers related to stigma, health literacy and motivation to discuss mental health, but have not been explored since this was not the main focus of this study. Future case studies might take these limitations into account to expand the insights acquired within this study.

### Clinical implications

An important implication for clinical practice is that healthcare providers, i.e. ophthalmologists, general practitioners and low vision service workers, should be aware of potential limitations adults with VI or blindness experience in recognizing and discussing mental health problems. In addition, they should understand their influence on the acknowledgement of and willingness to discuss mental health issues in visually impaired and blind adults. GPs’ knowledge on VI and overall healthcare providers’ knowledge on the impact of VI on mental health should be increased. Moreover, standard procedures could be introduced, with a screening instrument as a routine part of care. This might facilitate healthcare providers to start a conversation about the impact of VI on mental health. Finally, healthcare providers could actively provide information about depression and anxiety (psychoeducation), in a way that is suitable for visually impaired and blind adults (verbally, digitally or in Braille) to increase health literacy and reduce self-stigma.

## Conclusions

This study has revealed important factors related to the detection and discussion of depression and anxiety in visually impaired and blind adults. The results suggest that an increased vulnerability of adults with VI or blindness, concerning difficulty acknowledging both their VI and mental health problems, low health literacy, difficulty of attributing symptoms to the right impairment and reluctance to discuss symptoms, complicates recognizing and discussing mental health problems. Both the social support system and healthcare providers can play an important role in eliminating these barriers. Insights from this study could facilitate training for healthcare providers to improve detection and clinician-patient communication about depression and anxiety in adults with VI or blindness. Ultimately, this might improve quality of care and subsequently the quality of life of visually impaired and blind adults.

## Supplementary Information

**Additional file 1.** Interview guideline.

## Data Availability

The data that support the findings of this study are available on reasonable request from the corresponding author. The data are not publicly available due to their containing information that could compromise the privacy of research participants. Interview guide is included as supplementary material.

## Abbreviations

GP: General practitioner.
SD: Standard deviation.
VI: Visual impairment.
