# Supplementary material for: Barriers and facilitators to recognize and discuss depression and anxiety experienced by adults with vision impairment or blindness: a qualitative study
Source: BMC Health Serv Res. 2021 Jul 28;21:749. doi: 10.1186/s12913-021-06682-z (PMC8317369; doi:10.1186/s12913-021-06682-z)
Supplement: Supplementary file 1 — Additional file 1. Interview guideline. [file 12913_2021_6682_MOESM1_ESM.docx]

# Appendix 1. Interview guideline

In the beginning of the interview, the interviewer did not use the words *depression* and *anxiety*. Instead the interviewer used phrases, such as ‘not feeling like yourself’, ‘having a low mood’, ‘feeling anxious’ or ‘not being in a good mood’. If the client used words like depression and anxiety themselves, the interviewer switched to using these words.

**1. Awareness related factors**

- How did you recognize that you were not feeling like yourself? (prompt: What symptoms did you experience? When did you recognize you were not feeling like yourself?)
- What or who helped you to recognize you were not feeling like yourself?
- What made recognizing your symptoms more difficult?
- What did healthcare providers do to help you recognize your symptoms?
- What could have helped you to recognize your symptoms (more easy)?
- When could you acknowledge you were not feeling like yourself? (prompt: How did you notice?)
- What or who helped you to acknowledge you were not feeling like yourself?
- What made acknowledging your symptoms more difficult?
- What did healthcare providers do to help you acknowledge your symptoms?
- What could have helped you to acknowledge your symptoms (more easy)?
- What did you do when you acknowledged your symptoms? (prompt: What was your reason to (not) do something about it?)

**2. Discussing symptoms**

- Which healthcare provider(s) did you discuss your symptoms with?
- What made you decide to discuss your symptoms with this/these healthcare provider(s)? (prompt: How did healthcare providers influence your decision?)
- Who started the conversation about your system? (Prompt: When and how did this happen?)

**3. Motivational factors**

- What was your reason to discuss your symptoms with a healthcare provider?
- What was your reason, if you had any, to not discuss it?
- What helped, or could have helped, you to discuss your symptoms with a healthcare provider?
- What limited, or could have limited, you to discuss your symptoms with a healthcare provider?
- What did your social support system think about discussing your symptoms? (Prompt: How has this affected you?)
- How did healthcare providers influence your decision to on discuss your symptoms?
- What did you think of your skills and possibilities to discuss symptoms?

**4. Recommendations**

- What should remain the same, and what would you like to change, to improve the ability of adults with visual impairment to discuss symptoms with a healthcare provider? (Prompt: What is the healthcare provider’s role?)
